# Anti-staphylococcal activity, antibiotic-resistance modulation effects and action of *Harungana madagascariensis* (Hypericaceae) fruit extracts on the antioxidant system of multidrug-resistant *Staphylococcus aureus*

**DOI:** 10.1371/journal.pone.0329771

**Published:** 2025-08-07

**Authors:** Brenda Ngueffo Tiwa, Aimé Gabriel Fankam, Céline Brinda Sonfack, Richard Mouozong, Michael Francis Kengne, Armelle Tsafack Mbaveng, Victor Kuete

**Affiliations:** Department of Biochemistry, University of Dschang, Dschang, West, Cameroon; University of Buea, CAMEROON

## Abstract

Global public health is facing a real challenge due to infections caused by multidrug-resistant bacteria. Among these bacteria, *Staphylococcus aureus* is known to rapidly develops antibiotic resistance. This study aimed to evaluate the antibacterial potential of *Harungana madagascariensis* fruit extracts and their effects on the antioxidant system of multidrug-resistant *Staphylococcus aureus*. Moreover, the extracts were evaluated for their antibiotic-resistance modulation effects against some multidrug-resistant *Staphylococcus aureus*. The antibacterial activity of the extracts and their effect in combination with antibiotics were assessed using the micro-dilution method. The catalase activity was assessed by measuring the height of foam, whereas the lipid peroxidation was carried out through spectrophotometric quantification of malondialdehyde. The phytochemical analysis of extracts was carried out using qualitative and quantitative standard assays. The tested extracts showed antibacterial activities, with minimum inhibitory concentrations ranging from 32 to 2048 μg/mL. The most active extract (hexane extract) has inhibited the catalase activity and induced the lipid peroxidation in *S. aureus* DO18SA, indicating its ability to interact with the antioxidant system of the bacteria. Moreover, the dichloromethane/methanol extract increased by 2–128-fold the activity of levofloxacin, ampicillin, and cefotaxime against selected multidrug-resistant *S. aureus*. It also showed synergistic effect with cefotaxime against D051SA. Alkaloids, triterpenes, and phenols were detected in all the extracts, whereas the other phytochemical classes were selectively distributed. The methanol extract had the highest phenolic content (142.20 ± 16.75 mg GAE/g of extract). Overall, the findings of this study suggest that extracts of *Harungana madagascariensis* fruits could be valuable sources of new agents for treating multidrug-resistant *Staphylococcus aureus* infections.

## Introduction

One of the greatest advances made in medicine was the discovery of antibiotics, which has led to the saving of millions of lives [1]. Therefore, the overuse and misuse of these antibiotics make bacteria develop resistance to them, leading to an increase in nosocomial infections and therapeutic failures [2]. In the United States, antibiotic resistance causes around 2.8 million infections and 35,000 deaths a year [3]. The prevalence of multidrug-resistant (MDR) bacteria in Africa is high, with some studies reporting rates of up to 70% in hospitalised patients [4].

*Staphylococcus aureus* (*S. aureus*) is a human bacterial pathogen belonging to the genus *Staphylococcus* and characterized as Gram-positive cocci [5]. It is an opportunistic bacterium that is responsible for the majority of community and hospital-acquired infections such as, septicemia, endocarditis, and cutaneous infections [5,6]. Under pathogenic conditions, *S. aureus* is very difficult to treat due to its strong ability to produce virulence factors, to rapidly colonize the host, and to develop drug resistance mechanisms [7]. *S. aureus* infections caused approximately 1.1 million deaths globally in 2019 [8]. This is mainly attributed to its ability to develop resistance to multiple antibiotics such as β-lactams, aminoglycosides, macrolides, lincosamides, fluoroquinolones, chloramphenicol, sulfonamides, streptomycin, and tetracycline [9,10]. This is possible through multiple means, such as overexpression of efflux pumps, β- lactamases’ production, reduced porin expression, and mutation of quinolone targets [11,12]. Consequently, *S. aureus* has been placed on the World Health Organization (WHO) priority class bacteria list for the development of new antimicrobial agents. It is therefore very important to find new strategies to fight infections due to MDR *S. aureus*.

Reactive oxygen species (ROS) such as superoxide anion (O^2-^), hydroxyl radical (OH·), and hydrogen peroxide (H_2_O_2_) induce oxidative damage to the cellular macromolecule and may lead to cellular metabolic dysfunction. As one of the defence mechanisms, bacteria use enzymes such as catalases, which play a significant role in protecting bacteria from oxidative stress caused by hydrogen peroxide [13]. Moreover, ROS induce lipid peroxidation that causes changes in the membrane structure by altering its fluidity and damaging the membrane integrity [14]. So, botanicals or phytochemicals targeting the antioxidant system of the bacteria may serve as good candidates against MDR bacteria.

Medicinal plants, which are used by around 80% of the world’s population to treat various health problems, can be a good candidate for the discovery of new treatment options of microbial infections [15]. In fact, plants synthesize a wide variety of secondary metabolites, such as phenols, flavonoids, terpenoids, tannins, and alkaloids, endowed with antimicrobial properties [16,17].

*Harungana madagascariensis* Lam. ex Poir., belongs to the Hypericaceae family and is widely distributed in Africa [18]. It is used in African traditional medicine to treat many human diseases, including diarrhoea, dysentery, gonorrhoea, typhoid fever, jaundice, haemorrhoids, leprosy, anaemia, postpartum bleeding, and skin and heart problems. Its chemical evaluation has shown that different organs of *H. madagascariensis* contained phytochemicals such as anthranoids, anthraquinones, xanthones, flavonoids, triterpenoids, steroids, and alkaloids [18,19]. Moreover, botanicals and phytochemicals from *H. madagascariensis* have been reported to have many pharmacological properties, including antimicrobial, antioxidant, anti-sickling, and antiproliferative activities [20–25]. The leaf, bark, and root extracts of *H. madagascariensis* have been reported for their *in vitro* antibacterial activities against various pathogens. Therefore, no related activities reported for its fruits are accessible in the literature. So, this work aimed to evaluate the antibacterial potential of *H. madagascariensis* fruit extracts and their effects on the antioxidant system of MDR *S. aureus*. Moreover, these extracts were evaluated for their antibiotic-resistance modulation effects against some MDR *S. aureus* isolates.

## Materials and methods

### Plant material and extraction

*H. madagascariensis* (Hypericaceae) was collected in January 2024 at Dschang, West Cameroon. The plant sample was identified at the National Herbarium of Yaoundé, Cameroon, where the voucher has been deposited under the registration number 43848/HNC.

Air-dried fruits of *H. madagascariensis* were powdered and macerated in hexane, methanol, and the mixture dichloromethane/methanol (1:1) for 48 h at room temperature. After filtration on Whatman filter paper No. 1, each organic filtrate was concentrated using a rotary evaporator under reduced pressure to obtain the crude extracts. The residual solvent was removed by drying the crude extracts at 40°C in an oven. The extracts were then kept at 4°C until further use.

### Preliminary phytochemical screening of the extracts

#### Qualitative phytochemical screening.

In order to detect the different classes of secondary metabolites that were most likely to be responsible for the biological activities, a qualitative phytochemical screening was performed on the extracts as previously described [26,27].

#### Determination of the total phenolic content.

The total phenolic content (TPC) of the extracts was determined spectrophotometrically using the Folin-Ciocalteu reagent [28]. The reaction mixture consisted of 0.02 mL extract (2 mg/mL), 1 mL Folin-Ciocalteu reagent, and 0.8 mL 20% sodium carbonate solution. The mixture was stirred and incubated at 37°C for 30 min, then the absorbance was measured at 765 nm. The extract was replaced with distilled water in the control tubes. Results were expressed as milligrams of gallic acid equivalents per gram of extract (mg GAE/g). Each sample was assayed three times.

### Evaluation of the antibacterial activity of extracts

#### Bacteria and culture conditions.

In this study, fourteen clinical MDR isolates (D009SA, D018SA, D020SA, D021SA, D031SA, D047SA, D049SA, D050SA, D051SA, D052SA, D057SA, D060SA, D074SA, and D094SA) and a reference strain (ATCC25923) of *S. aureus* were utilized. The reference strain (ATCC25923) was from American Type Culture Collection. The features of these pathogenic bacteria are presented in the supporting information (S1 Table). Mueller-Hinton agar (ReadyMED®, USA) was used to culture and maintain the bacteria, whereas Mueller Hinton broth (MHB) (ReadyMED®, USA) was used for the preparation of the bacterial inoculum and to carry out the antibacterial assays.

#### Determination of the minimum inhibitory and bactericidal concentrations.

The minimum inhibitory concentration (MIC) of extracts was determined using INT colorimetric assay [29] with slight modifications [30]. Briefly, 100 µL of extract or reference antibacterial drug, dissolved in 10% dimethyl sulfoxide (DMSO, BDH Chemicals Ltd, Poole, England)/MHB, were serially diluted two-fold in a 96-well sterile microplate. Thereafter, 100 μL of inoculum (1.5 × 10^6^ CFU/mL) were added in each well, and the plates were covered with a sterile plate sealer and incubated at 37°C for 18 h. Wells containing inoculum without extract served as growth control. The MIC, defined as the lowest sample concentration that prevented the growth of the bacteria, was then detected after the addition of 30 μL of 0.2 mg/mL p-iodonitrotetrazolium chloride (INT, Loba Chemie Pvt Ltd, Mumbai, India) in each well of the plates, followed by incubation at 37°C for 30 min. Viable bacteria reduced the yellow dye to pink. The minimum bactericidal concentrations (MBC) of each extract was determined by adding 50 μL aliquots of the preparations which did not show any growth after incubation during MIC determination, to 150 μL of MHB in a new 96-well microplate. These preparations were incubated at 37°C for 48 h. MBC was considered as the lowest concentration of sample that prevented the color change of the medium after the addition of INT [30]. Each experiment was carried out in duplicate and repeated three times.

The antibacterial activity of extracts was interpreted as followed based on the most recent cut-off values: Outstanding activity if MIC < 8 μg/mL; excellent activity if 8 < MIC ≤ 40 μg/mL; Very good activity if 40 < MIC ≤ 128 μg/mL; good activity if 128 < MIC ≤ 320 μg/mL; average activity if 320 < MIC ≤ 625 μg/mL; weak activity if 625 < MIC ≤ 1024 μg/mL and not active if MIC >1024 μg/mL [31]. Moreover, extract was considered bactericidal, if MBC/MIC ≤ 4 and bacteriostatic if MBC/MIC > 4 [32].

#### Assessment of the antibiotic-resistance modifying effects of extracts.

The antibiotic modulation effect of extracts was performed as previously described [33,34]. The MICs of ciprofloxacin, levofloxacin, streptomycin, ampicillin, ceftriaxone, cefotaxime, and vancomycin (Sigma-Aldrich) were determined in the presence and absence of a sub-inhibitory concentration (MIC/8) of different extracts. The modulation factor (MF), defined as the ratio between the MIC of the antibiotic alone and that of the antibiotic in the presence of the extract, was calculated. MF ≥ 2 has been set as the threshold for biological significance of the antibiotic modulation effect [35,36]. Each assay was performed in duplicate and repeated thrice.

#### Synergy assay.

The synergy effect of the combination of antibiotics (ampicillin and cefotaxime) and Cl_2_CH_2_/ MeOH extract was investigated using the checkerboard broth micro-dilution method [37]. In a 96-well sterile microplate, 100 µL of antibiotic were introduced in the first well of each column followed by two-fold serial dilutions of the antibiotic from row A to G. Then, 100 µL of extract were introduced in the first well of each row, followed by two-fold serial dilutions from column 1–10. One hundred (100) µL of inoculum were then added to all wells except the last four wells of colon 12 and the last well of column 11, which were used as controls. The microplates were then incubated at 37^°^C, and MIC was determined after 18 h of incubation as previously described. The fractional inhibitory concentration index (ƩFIC) for all the combinations was determined using the following formula:


$$\sum FIC=\frac{\;MIC\;of\;extract\;in\;combination}{MIC\;of\;extract\;alone}+\;\frac{\;MIC\;of\;antibiotic\;in\;combination}{MIC\;of\;antibiotic\;alone}$$

The interaction was considered as: synergy (ƩFIC ≤ 0.5); additivity (0.5 < ƩFIC ≤ 1); indifferent if (ΣFIC >1–2); and antagonism (ƩFIC > 2) [38].

### Evaluation of the effect of the hexane extract on the antioxidant system *S. aureus*

#### Effect of the hexane extract on catalase activity of *S. aureus.*

The effect of the most active extract (hexane extract) on catalase activity of *S. aureus* (D018SA) was carried out as previously described [39]. Briefly, fresh colonies of *S. aureus* were inoculated in 5 mL of sterile MHB and incubated at 37°C for the whole night. Likewise, overnight cultures of *S. aureus* were cultured in a medium containing 1 mL of hexane extract (MIC and 2xMIC) or polymyxin B (MIC) and 4 mL of MHB. Then, 100 µL of each sample’s aliquot were put into a test tube in addition to 100 µL of 1% Triton X-100 and 100 µL of 30% (*v/v*) H_2_O_2_. Once the tubes were well mixed, they were kept at room temperature for 5 minutes. The foam’s height unchanged for fifteen minutes, was then measured in cm with a ruler and compared to the untreated D018SA.

#### Lipid peroxidation assay.

The level of lipid peroxidation was performed by measuring the concentration of malondialdehyde (MDA) using thiobarbituric acid [40]. Briefly, fresh colonies of *S. aureus aureus* (D018SA) were cultured overnight in 4 mL of MHB containing 1 mL of hexane extract (MIC and 2xMIC) or polymyxin B (MIC). Thereafter, 1 mL of the test culture, 1 mL of thiobarbituric acid, and 1 mL of trichloroacetic acid were introduced in a screw-cap test tube, mixed well, and heated to 100°C for 10 minutes in a water bath. After the test tubes were allowed to cool, they were centrifuged for 20 minutes at 5000 rpm. The supernatants were collected, and their absorbances were measured at 535 nm against the control. The MDA concentration (nM) was calculated based on the millimolar absorbance coefficient (E_0_ = 156 cm^−1^.mM^−1^) using the equation shown below:


$$MDA\;\left(nM\right)=\frac{Sample\;absorbance\;+\;Control\;absobance}{E_{0}}\times 10^{6}$$

## Statistical analysis

The data for TPC, catalase activity, and the MDA concentration were presented as the mean with the standard deviation (Mean ± SD) from three replicates. One-way analysis of variance (ANOVA) followed by Tukey’s multiple comparison test was employed to compare the means. The statistical analysis and graphs were done with GraphPad Prism for Windows, version 5.0.1. Results were considered significant if **p* *< 0.05.

## Results

### Phytochemical composition of extracts

#### Qualitative phytochemical composition.

The results of the phytochemical screening showed that all the tested extracts contain alkaloids, triterpenes, and phenols. Moreover, flavonoids, saponins, and anthraquinones were detected in dichloromethane/methanol (Cl_2_CH_2_/MeOH) and methanol (MeOH) extracts. All the seeking phytochemicals were present in the MeOH extract (Table 1).

**Table 1:** Qualitative chemical composition of *H. madagascariensis* fruit extracts.

Extracts	Phytochemical classes						
	Alkaloids	Flavonoids	Saponins	Tannins	Triterpenes	Phenols	Anthraquinones
Hexane	**+**	—	—	—	**+**	+	—
Cl_2_CH_2_/MeOH	**+**	**+**	+	—	**+**	**+**	**+**
MeOH	**+**	**+**	+	**+**	**+**	**+**	**+**

Cl_2_CH_2_/MeOH: Dichloromethane/methanol. MeOH: Methanol. (+): Present; (—): Absent.

#### Total phenolic content of extracts.

**Fig 1:**
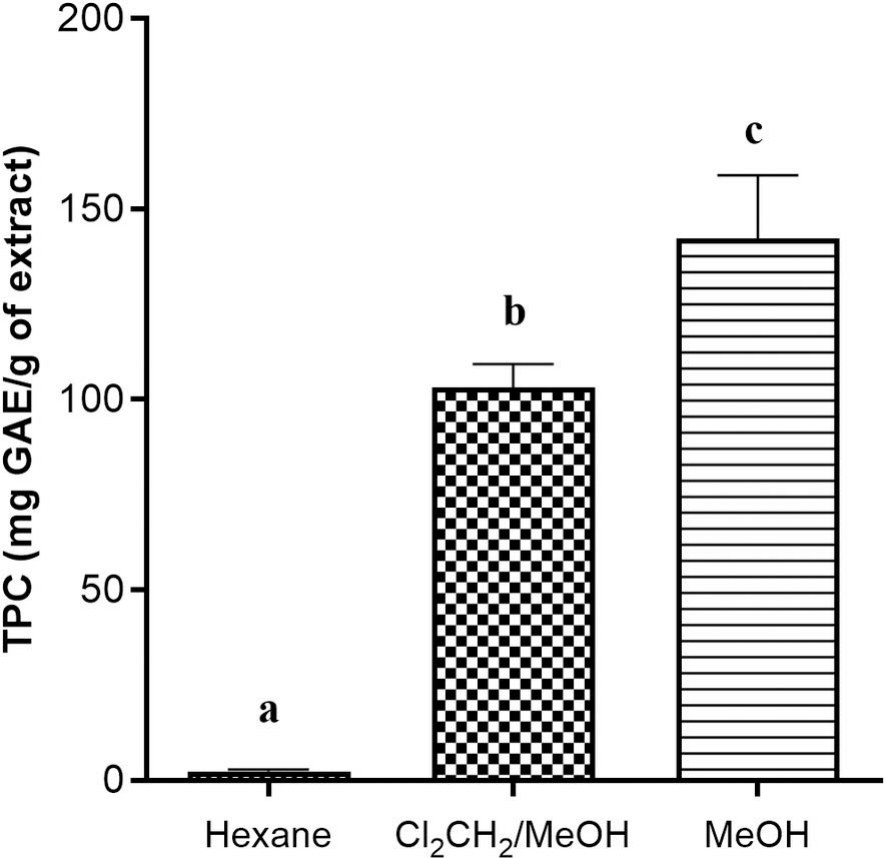
Total phenolic content of *H. madagascariensis* fruit extracts. TPC: Total phenolic content; GAE: gallic acid equivalents. Cl_2_CH_2_/MeOH: Dichloromethane/methanol. MeOH: Methanol.

This study evaluated the TPC of the tested extracts. The results showed that the MeOH extract of *H. madagascariensis* fruits presented the highest TPC (142.20 ± 16.75 mg GAE/g extract), followed by Cl_2_CH_2_/MeOH extract (103.27 ± 6.03 mg GAE/g). Indeed, the hexane extract showed the lowest phenolic content (2.40 ± 0.42 mg GAE/g of extract) (Fig 1).

### Antibacterial activity of *H. madagascariensis* fruit extracts

#### MIC and MBC of extracts.

The data summarized in Table 2 present the MIC and MBC of the tested extracts against pathogenic *S. aureus*. The results showed that these extracts had antibacterial activity with MIC ranging from 32 to 2048 µg/mL. The MeOH extract displayed inhibitory activity on all the tested isolates (100%), whereas the hexane and Cl_2_CH_2_/MeOH extracts have inhibited the growth of 14/15 (93.33%) of the tested bacteria. The hexane extract appears as the most active extract. It displayed excellent activity against *S. aureus* D018SA (MIC = 32 µg/mL) ATCC25923, and D020SA (MIC = 64 µg/mL). It was more active on *S. aureus* D018SA (MIC = 32 µg/mL) than streptomycin, which was used as reference antibacterial drug (MIC > 256 µg/mL). Globally, MBCs of the tested extracts were above 2048, and those ≤ 2048 µg/mL were detected on at least 4/15 of the tested bacteria. The MBC/MIC ratio of all the tested extracts was less than 4 on at least one strain. This was mostly observed with Cl_2_CH_2_/MeOH extract (7/15 of the tested *S. aureus*). Therefore, the MBC/MIC ratio was more than 4 for the hexane extract on the most sensitive strains (ATCC25923, D018S, and D020SA).

**Table 2:** MIC, MBC and MBC/MIC of *H. madagascariensis* fruit extracts and streptomycin against *Staphylococcus aureus.*

*S. aureus*	MIC (µg/mL), MBC (µg/mL) and MBC/MIC									Streptomycin		
	Hexane			Cl_2_CH_2_/MeOH			MeOH					
	MIC	MBC	MBC/MIC	MIC	MBC	MBC/MIC	MIC	MBC	MBC/MIC	MIC	MBC	MBC/MIC
ATCC25923	**64**	1024	16	512	>2048	—	1024	>2048	—	32	>256	—
D009SA	1024	>2048	—	2048	>2048	—	2048	>2048	—	>256	>256	—
D018SA	**32**	1024	16	1024	2048	2	2048	>2048	—	>256	>256	—
D020SA	**64**	512	8	128	1024	8	128	2048	16	64	>256	—
D021SA	1024	>2048	—	1024	>2048	—	2048	>2048	—	>256	>256	—
D031SA	1024	>2048	—	>2048	>2048	—	1024	2048	2	128	>256	—
D047SA	512	>2048	—	1024	>2048	—	1024	2048	2	>256	>256	—
D049SA	2048	>2048	—	1024	>2048	—	1024	>2048	—	>256	>256	—
D050SA	1024	>2048	—	256	2048	2	1024	>2048	—	64	>256	—
D051SA	2048	>2048	—	2048	2048	1	1024	>2048	—	>256	>256	—
D052SA	2048	>2048	—	512	2048	4	2048	>2048	—	>256	>256	—
D057SA	>2048	>2048	—	512	2048	4	1024	>2048	—	128	>256	—
D060SA	256	1024	2	1024	2048	2	2048	>2048	—	64	>256	—
D074SA	1024	>2048	—	1024	>2048	—	1024	>2048	—	>256	>256	—
D094SA	2048	>2048	—	1024	1024	1	512	1024	2	>256	>256	—

Cl_2_CH_2_/MeOH: Dichloromethane/methanol; MeOH: Methanol; MIC: Minimum Inhibitory Concentration; MBC: Minimum Bactericidal Concentration; —: MBC/MIC not determined; Values in bold indicate excellent activity (8 < MIC ≤ 64 µg/mL).

### Antibiotic-resistance modulation effects of extracts

In order to select the extracts likely to potentiate the antibiotic activity, a preliminary experiment carried out against D094SA (S2 Table) has allowed us to select the CH_2_Cl_2_/MeOH extract as a potential antibiotic modulator. This extract improved by 2–128-fold the activity of the tested antibiotics on MDR *S. aureus*. This was observed with cefotaxime (80%), vancomycin and levofloxacin (60%), ceftriaxone and streptomycin (40%), and ciprofloxacin and ampicillin (20%) in the presence of CH_2_Cl_2_/MeOH extract (Table 3).

**Table 3:** Antibiotic potentiating effects of CH_2_Cl_2_/MeOH extract of *H. madagascariensis* fruits.

		Bacteria, MIC (μg/mL) and modulation factor (in bracket)					AME (%)
Antibiotics	Extract	D020SA	D049SA	D051SA	D074SA	D094SA	
Ciprofloxacin	0 MIC/8	64 128 (0.5)	8 8 (1)	32 8 (**4**)	16 16 (1)	128 256 (0.5)	20
Levofloxacin	0 MIC/8	32 32 (1)	8 8 (1)	8 2 (**4**)	16 4 (**4**)	64 16 (**4**)	60
Streptomycin	0 MIC/8	> 256 128 (**>2**)	256 256 (1)	128 32 (**4**)	> 256 > 256 (—)	> 256 > 256 (—)	40
Ampicillin	0 MIC/8	> 256 256 (>1)	> 256 > 256 (—)	256 4 (**128**)	> 256 > 256 (—)	> 256 > 256 (—)	20
Ceftriaxone	0 MIC/8	64 256 (0.25)	256 256 (1)	32 16 **(2)**	256 64 (**4**)	64 64 (1)	40
Cefotaxime	0 MIC/8	32 8 (**4)**	128 128 (1)	64 16 (**4**)	256 64 (**4**)	128 32 (**4**)	80
Vancomycin	0 MIC/8	32 16 (**2**)	16 2 (**8**)	> 256 > 256 (—)	64 8 (**8**)	> 256 > 256 (—)	60

MIC: Minimum Inhibitory Concentration; AME: Antibiotic-resistance modulation effect; **—**: MF not determined; values in bold represent modulation factors ≥ 2.

### Interactions between antibiotics and CH_2_Cl_2_/MeOH extract

The interaction effects between antibiotics and CH_2_Cl_2_/MeOH extract were evaluated through the determination of the fractional inhibitory concentration index (ΣFIC). The results showed synergy (ΣFIC = 0.375) for the combination of cefotaxime with CH_2_Cl_2_/MeOH extract and additivity (ΣFIC = 0.56) for the combination of ampicillin with CH_2_Cl_2_/MeOH extract (Table 4).

**Table 4:** Iinteraction effects between antibiotics and CH_2_Cl_2_/MeOH extract against *S. aureus* D051SA.

Antibiotics	MIC alone		MIC combination		ƩFIC	Interaction
	ATB	Extract	ATB	Extract		
Cefotaxime	64	2048	8	512	**0.375**	**Synergy**
Ampicillin	256	2048	16	1024	0.56	Additivity

ATB: Antibiotic, ƩFIC: Fractional Inhibitory Concentration index; MIC: Minimum Inhibitory Concentration.

### Effect of the active extract on the antioxidant system *S. aureus*

#### Effect of the hexane extract on catalase activity of D018SA.

**Fig 2:**
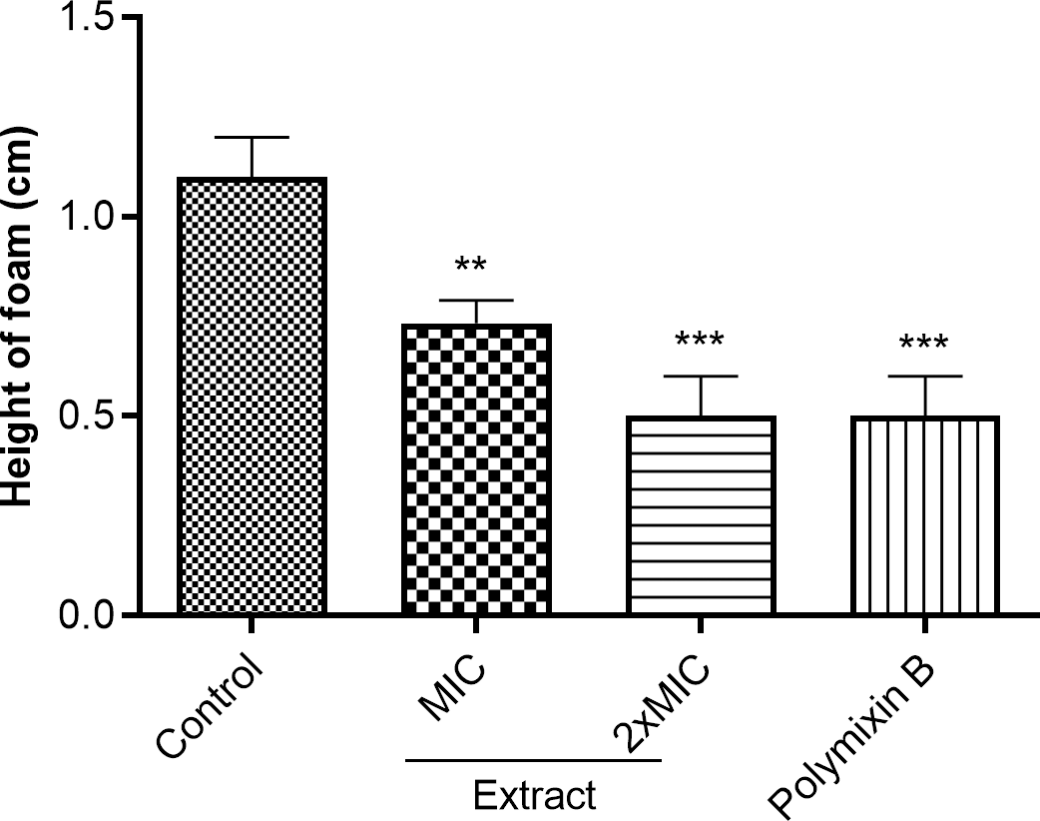
Catalase activity in extract treated DO18SA. MIC: Minimal inhibitory concentration. The MIC of polymyxin B and extract against D018SA were 16 and 32 µg/mL, respectively. ** (p < 0.01); *** (p < 0.001).

The catalase activity of D018SA was determined by measuring the height of foam. In untreated cells, the height of foam was 1.05 ± 0.07 cm, whereas in extract-treated D018SA, it was 0.70 ± 0.14 cm and 0.65 ± 0.07 cm at MIC and 2xMIC, respectively (Fig 2). These results indicate a significant (p < 0.05) inhibition of the catalase activity of D018SA by the hexane extract.

### Effect of the active extracts on D018SA lipid peroxidation

**Fig 3:**
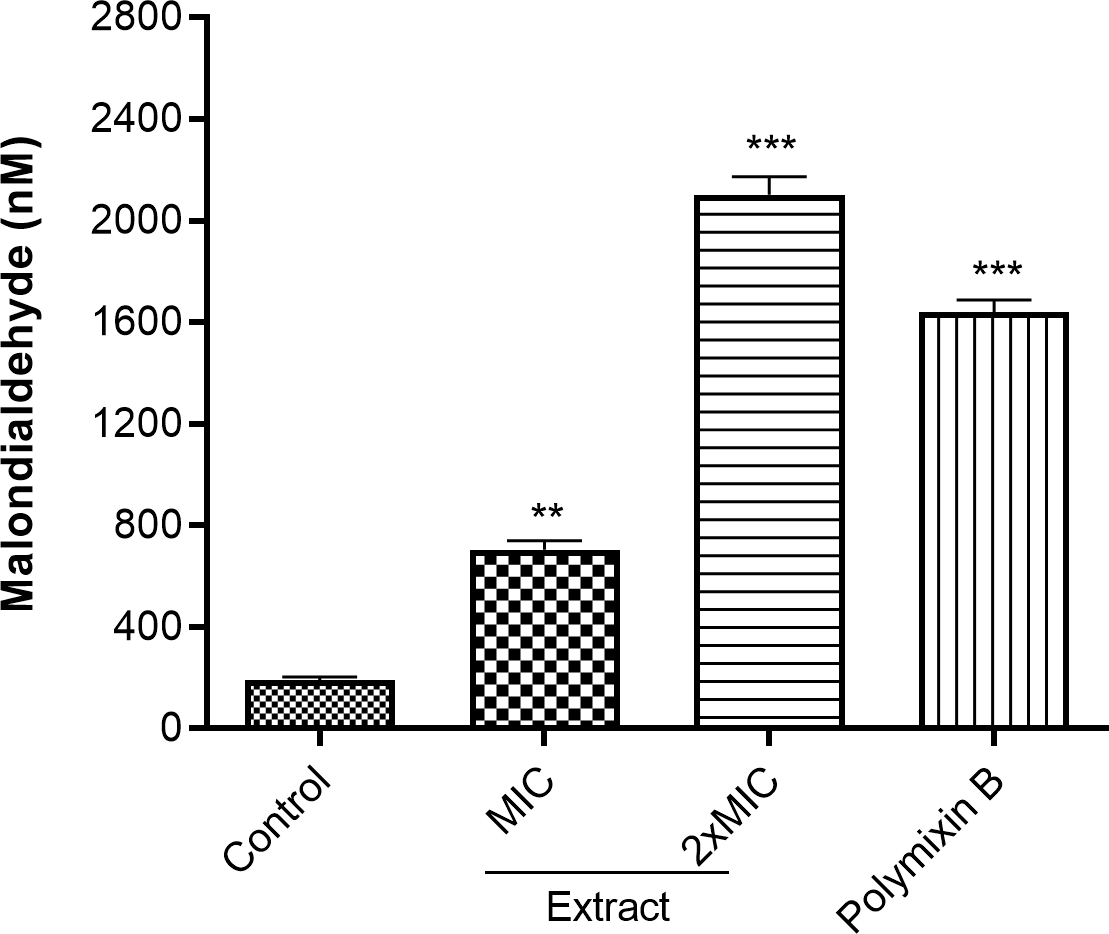
Malondialdehyde production in extract treated D018SA. MIC: Minimal inhibitory concentration; The MIC of polymyxin B and extract against D018SA were 16 and 32 µg/mL, respectively. ** (p < 0.01); *** (p < 0.001).

The lipid peroxidation activity (expressed in terms of malondialdehyde concentration) was evaluated in extract-treated D018SA. The MDA concentration was 189.27 ± 13.35 nM for control, 704.85 ± 35.86 nM and 2101.30 ± 70.74 nM in extract-treated *S. aureus* D018SA, at MIC and 2xMIC, respectively. Moreover, the effect of extract (2xMIC) was high compared to that of polymyxin B (1639.86 ± 46.97 nM) used as standard (Fig 3). These results show that this extract has significantly (p < 0.05) induced the lipid peroxidation in D018SA.

## Discussion

The global spread of MDR pathogens is leading to the reduction in the arsenal of antibiotics, and therefore to the need for the development of new drugs with novel target mechanisms [41,42]. This study was aimed at investigating the antibacterial potential of *Harungana madagascariensis* fruit extracts and their effects on the antioxidant system of multidrug-resistant *S. aureus*. Moreover, the extracts were evaluated for their antibiotic-resistance modulation effects against some MDR isolates.

Medicinal plants may contain antimicrobial compounds that are used to treat infectious disorders [16,43]. In the first part of this study, we have evaluated the anti-staphylococcal activity of *H. madagascariensis* fruit extracts against some clinical MDR isolates. The findings have shown that the tested extracts presented varied antibacterial potentials. This could be attributed to the difference in phytochemical composition observed between the extracts [44]. The difference observed may also be due to the different solvent used for the extraction [45]. According to the cut-off points of MICs established by Wamba *et al*. [31], the hexane fruits’ extract showed excellent activity (8 < MIC ≤ 64 µg/mL) against some of the tested bacteria. Moreover, its MBC/MIC ratio was more than 4 on the most sensitive strains (ATCC25923, D018S, and D020SA), suggesting that it has a bacteriostatic effect [32]. The result of this work is in agreement with previous studies that have demonstrated the antibacterial activity of leaves, bark, and roots of *H*. *madagascariensis* against other bacteria species [20,21]*.* For instance, Tankeo *et al.* (2016) have shown that the methanol extract of *H*. *madagascariensis* bark had excellent activity (MIC ≤ 8 µg/mL) against some Gram-negative strains, including *Escherichia coli* ATCC10536 and W3110. They have identified ferruginin A, an anthranoid, as the possible active ingredient of that extract [20]. Moreover, Tegaboue *et al*. (2021) have shown that leaves, bark, and roots of *H*. *madagascariensis* have excellent to moderate activity on some clinical bacteria, including *S. aureus* [21]. Astilbin, a flavone isolated from the ethyl acetate extract of *H. madagascacriensis* leaves, was also shown to have strong activity against *Staphylococcus epidermidis, Micrococcus luteus, Acinetobacter* sp.*,* and *Moraxella* sp. [46]. In this study, the hexane extract has presented the best activity, with MIC = 32 µg/mL on *S. aureus* D018 SA, and with MIC = 64 µg/mL against ATCC10536 and D020SA. This may be attributed to the presence of nonpolar compounds such as triterpenes or simple phenols detected in this extract. Taken together our findings, this study highlights the potential of *H*. *madagascariensis,* and especially its hexane fruit extract, as a candidate for the discovery of an anti-staphylococcal drug.

The discovery of innovative drugs with new modes of action could represent a promising solution to counteract the ongoing emergence and spread of resistant infections. These new drugs should be designed to escape preexisting mechanisms of resistance [42]. Herein, the effect of the hexane extract has been assessed on the D018SA antioxidant system. Many pathogens are known to produce catalase enzymes to protect themselves from hydrogen peroxide, a defense mechanism commonly used by the host’s immunity. Previous studies have reported that catalase-deficient mutant pathogens are more sensitive to oxidative stress and attack by the host immune system [47]. There is no literature available describing the mechanism of extracts of *H*. *madagascariensis* upon bacterial catalase activity. In this study, the hexane extract of *H*. *madagascariensis* fruits has inhibited the catalase activity of *S. aureus* D018SA. This could be due to the presence of simple phenols, such as thymol, carvacrol and other phenolic acids known to possess antibacterial properties and to inactivate bacterial enzyme systems as one of their potential modes of action [39]. The hexane extract has induced lipid peroxidation in *S. aureus* D018SA. This may be a consequence of the inhibition of catalase activity causing an increase of ROS, which may induce lipid peroxidation. In fact, it is known that catalase produced under oxidative stress conditions can prevent lipid peroxidation [48]. Therefore, when the catalase is nonfunctional or inhibited, ROS induce lipid peroxidation, which causes changes in the cell membrane structure by altering its fluidity and damaging the membrane integrity [14,49]. Moreover, the presence of triterpenes in the hexane extract may also explain these results. In fact, many reports have shown that triterpenes induce oxidative stress by reacting with bacterial membrane components such as lipids to produce ROS, which causes lipid peroxidation [50]. Inhibiting multidrug resistance mechanisms such as efflux pump expression, β-lactamase production, and biofilm formation is currently considered as a potential strategy to reduce the spread of resistance in bacteria [51–53]. Among the existing approaches, many researchers have reported that the combination of plant extracts with antibiotics could exhibit synergy effects against MDR bacteria, including *S. aureus* [35,54–56]. In this study, we have evaluated the combination effects of *H. madagascariensis* fruit extracts with eight commonly used antibiotics. It appeared that CH_2_Cl_2_/MeOH extract of *H. madagascariensis* fruit has increased by 2–128-fold the efficacy of levofloxacin, ampicillin, and cefotaxime on MDR *S. aureus*. It also showed a synergy effect (ƩFIC = 0.375) with cefotaxime against the *S. aureus* DO51SA. This may be attributed to the presence of secondary metabolites such as alkaloids, terpenoids, and flavonoids, which were detected in high proportions in this extract during this study. It has been demonstrated that these phytochemicals could inhibit the expression of efflux pumps [57,58] or β-lactamases [59,60], known as important resistance mechanisms in *S. aureus* [11,12].

## Conclusions

The findings of this study gave valuable insight into the potential usage of *H. madagascariensis* fruit extracts for treating microbial infections, especially those due to MDR *Staphylococcus aureus*. The study provided the first evidence of the antimicrobial-resistance modulation effect and the inhibition of the antioxidant system of *H. madagascariensis* fruit extracts against *Staphylococcus aureus*. It also provides scientific credence to the traditional uses of *H. madagascariensis* for managing microbial infections. So, these extracts could be considered as important sources of new agents to treat infections due to MDR *Staphylococcus aureus*. Therefore, more research on these extracts may be done regarding the identification of their active ingredients as well as their safety investigations.

## Supporting information

S1 Table Bacteria used and their features. (PDF)

S2 Table Results of the preliminary modulation essay of extracts at its sub-inhibitory concentration (MIC/8) against *S. aureus* D094SA. (PDF)
