# Correction: Anti-staphylococcal activity, antibiotic-resistance modulation effects and action of *Harungana madagascariensis* (Hypericaceae) fruit extracts on the antioxidant system of multidrug-resistant *Staphylococcus aureus*

**DOI:** 10.1371/journal.pone.0338020

**Published:** 2025-12-04

**Authors:** Brenda Ngueffo Tiwa, Aimé Gabriel Fankam, Céline Brinda Sonfack, Richard Mouozong, Michael Francis Kengne, Armelle Tsafack Mbaveng, Victor Kuete

The Copyright line for this article is incorrect. The correct copyright line is: © 2025 Tiwa et al. This is an open access article distributed under the terms of the Creative Commons Attribution License, which permits unrestricted use, distribution, and reproduction in any medium, provided the original author and source are credited.

In the Evaluation of the effect of the hexane extract on the antioxidant system S. aureus subsection of the Materials and Methods, there is an error in the equation under Lipid peroxidation assay. Please view the complete, correct equation here:


$$𝐌𝐃𝐀\;\left(𝐧𝐌\right)=\frac{𝐒𝐚𝐦𝐩𝐥𝐞\;𝐚𝐛𝐬𝐨𝐫𝐛𝐚𝐧𝐜𝐞\;-\;𝐂𝐨𝐧𝐭𝐫𝐨𝐥\;𝐚𝐛𝐬𝐨𝐫𝐛𝐚𝐧𝐜𝐞}{{𝐄}_{0}}\times 10^{6}$$

In Fig 2 and Fig 3, the label “Polymixin B” should have been “Polymyxin B”. Please see the correct Fig 2 and Fig 3 here.

**Fig 2:**
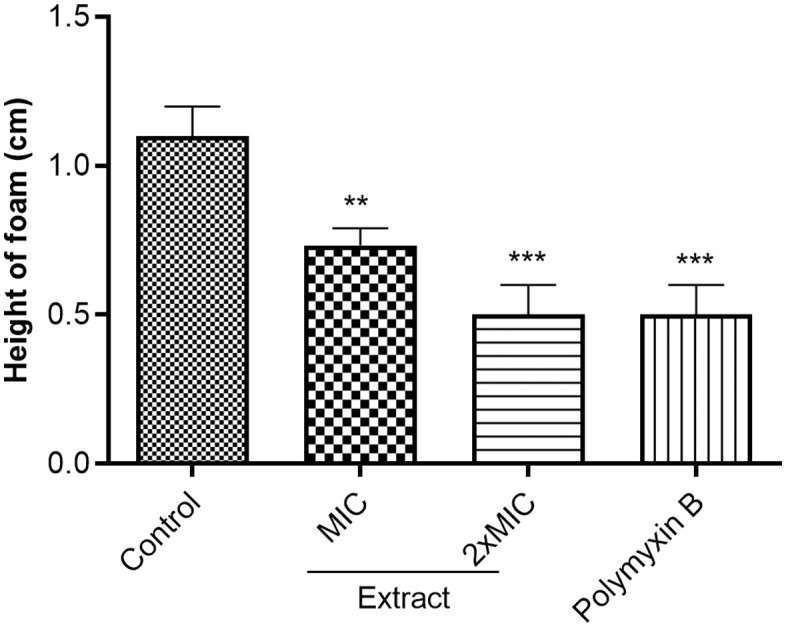
Catalase activity in extract treated DO18SA. MIC: Minimal inhibitory concentration. The MIC of polymyxin B and extract against D018SA were 16 and 32 µg/mL, respectively. ** (p < 0.01); *** (p < 0.001).

**Fig 3:**
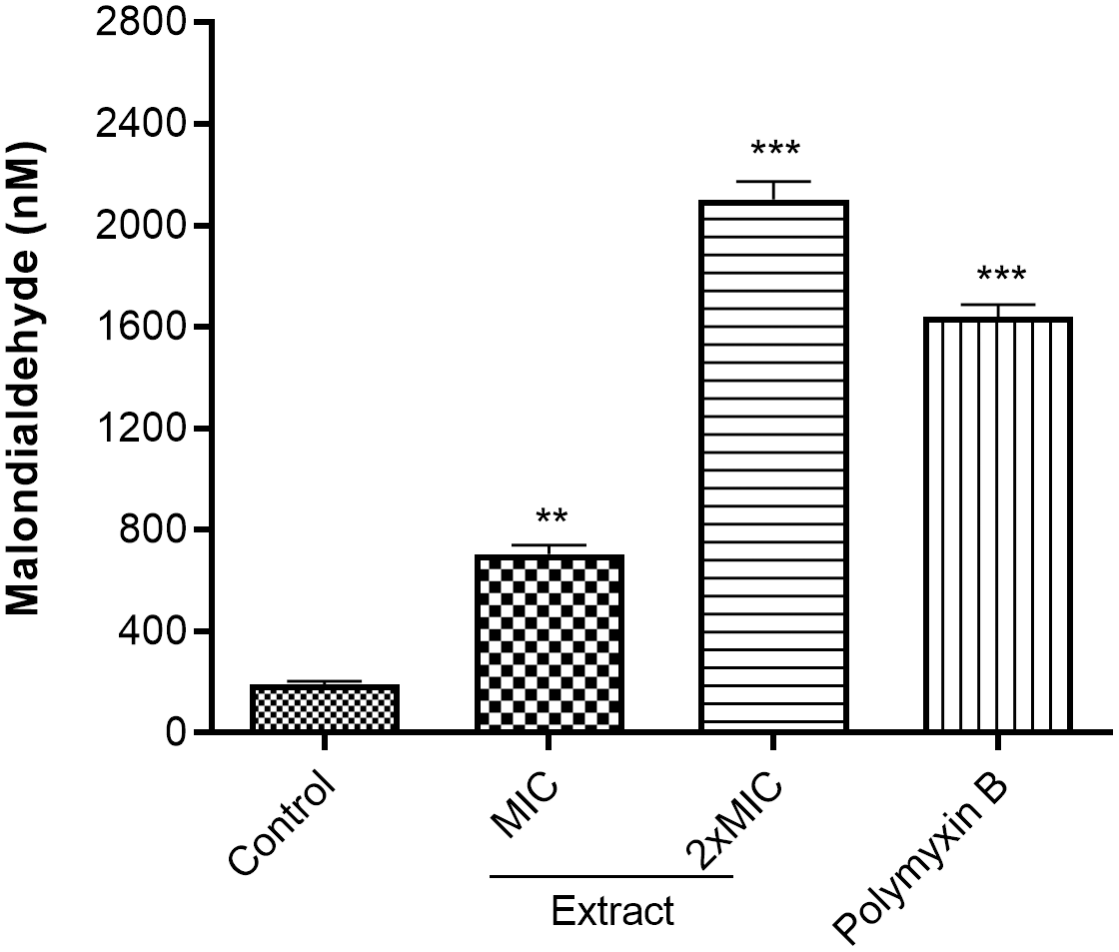
Malondialdehyde production in extract treated D018SA. MIC: Minimal inhibitory concentration; The MIC of polymyxin B and extract against D018SA were 16 and 32 µg/mL, respectively. ** (p < 0.01); *** (p < 0.001).
