# Supplementary material for: Anti-staphylococcal activity, antibiotic-resistance modulation effects and action of Harungana madagascariensis (Hypericaceae) fruit extracts on the antioxidant system of multidrug-resistant Staphylococcus aureus
Source: PLoS One. 2025 Aug 7;20(8):e0329771. doi: 10.1371/journal.pone.0329771 (PMC12331101; doi:10.1371/journal.pone.0329771)
Supplement: S1 Table — (PDF) [file pone.0329771.s001.pdf]

S1 Table. Bacteria used and their features.

Bacteria	Characteristics	References
ATCC25923	Bacterial strain American type culture collection	
D009SA	Clinical isolate : AMX^r, ERY^r, VAN^r,	(Kengne *et al*., 2024)
D018SA	Clinical isolate : AMX^r, CAZ^r, FOX^r, AMC^r, CXM^r ·COT^r ERY^r ,FUS^r,TET^r,OXA^r, VAN^r, NIT^r,	(Kengne *et al*., 2024)
D020SA	Clinical isolate : AMX^r, CAZ^r, FOX^r, AMC^r, CXM^r, COT^r ERY^r, VAN^r, AMK^r, CIP^r, OFX^r,	(Kengne *et al*., 2024)
D021SA	Clinical isolate : AMX^r, CAZ^r, FOX^r, VAN^r, OXA^r,	(Kengne *et al*., 2024)
D031SA	Clinical isolate : IMP^r, AMC^r, FOX^r, CXM^r, COT^r, ERY^r, TET^r, OXA^r, VAN^r	(Kengne *et al*., 2024)
D047SA	Clinical isolate : AMX^r, CAZ^r, CXM^r, COT^r, ERY^r ,TET^r, OXA^r, VAN^r	(Kengne *et al*., 2024)
D049SA	Clinical isolate : CAZ^r, FOX^r, AMC^r, CXM^r, COT^r, ERY^r, TET^r,OXA^r, VAN^r, NIT^r, CIP^r, OFX^r	(Kengne *et al*., 2024)
D050SA	Clinical isolate : AMX^r, CAZ^r, ERY^r, FUS^r, TET^r, OXA^r, VAN^r, CIP^r,	(Kengne *et al*., 2024)
D051SA	Clinical isolate : AMX^r, FOX^r, CXM^r, ERY^r, CIP, ^{r} OFX^r, FUS^r, TET^r, OXA^r, VAN^r, NIT^r	(Kengne *et al*., 2024)
DO52SA	Clinical isolate : AMX^r, CAZ^r, COT^r, ERY^r, FUS^r, TET^r, OFX^r, VAN^r	(Kengne *et al*., 2024)
D057SA	Clinical isolate : AMX^r, IMP^r, FOX^r, CIP, CXM^r, ERY^r, OFX^r, VAN^r	(Kengne *et al*., 2024)
D060SA	Clinical isolate : AMX^r, CAZ^r, COT^r, FUS^r, ERY^r,TET^r, OXA^r, VAN^r, NIT^r	(Kengne *et al*., 2024)
D074SA	Clinical isolate : AMX^r, CAZ^r, FOX^r, CXM^r, COT^r, ERY^r, AMK^r, TET^r, GEN^r, FUS^r,	(Kengne *et al*., 2024)
D094SA	Clinical isolate : AMX^r, CAZ^r, FOX^r, AMC^r, CXM^r, COT^r, ERY^r, OFX^r, CIP^r, FUS^r, TET^r, OXA^r, VAN^r, NIT^r,	(Kengne *et al*., 2024)

AMX: Amoxicillin, AMC: Amoxicillin -clavulanic acid, CAZ: Ceftazidime, FOX: Cefoxitin, AMK: Amikacin, GEN: Gentamicin, TET: Tetracycline, CIP: Ciprofloxacin, NIT: Nitrofurantoin, OFX: Ofloxacin, COT: Trimethoprim-sulfamethoxazole, FUS: Fusidic acid, ERY: Erythromycin; r: resistant.

References

Kengne MF, Mbaveng AT, Kuete V. Antibiotic Resistance Profile of *Staphylococcus aureus* in Cancer Patients at Laquintinie Hospital in Douala, Littoral Region, Cameroon. *Biomed Res Int.* 2024; 2024:5859068. Doi: 10.1155/2024/5859068.
