# Supplementary material for: Anti-staphylococcal activity, antibiotic-resistance modulation effects and action of Harungana madagascariensis (Hypericaceae) fruit extracts on the antioxidant system of multidrug-resistant Staphylococcus aureus
Source: PLoS One. 2025 Aug 7;20(8):e0329771. doi: 10.1371/journal.pone.0329771 (PMC12331101; doi:10.1371/journal.pone.0329771)
Supplement: S2 Table — (PDF) [file pone.0329771.s002.pdf]

S2 Table. **Results of the preliminary modulation essay of extracts at its sub-inhibitory concentration (MIC/8) against *S. aureus* D094SA.**

**Antibiotics**	**Extracts concentrations**	**Samples, MIC (µg/mL) and modulation factor (in bracket)**		
		**Hexane**	**Cl_2CH_2/MeOH**	**MeOH**
Ciprofloxacin	0	128	128	128
	MIC/8	256(0,5)	256 (0,5)	256 (0,5)
Levofloxacin	0	64	64	64
	MIC/8	16(**4**)	16 (**4**)	16 (**4**)
Streptomycin	0	> 256	> 256	> 256
	MIC/8	> 256 (—)	> 256 (—)	> 256 (—)
Ampicillin	0	> 256	> 256	> 256
	MIC/8	> 256 (—)	> 256 (—)	> 256 (—)
Ceftriaxone	0	> 256	> 256	> 256
	MIC/8	256 (>1)	> 256 (—)	> 256 (—)
Cefotaxime	0	128	128	128
	MIC/8	128 (1)	32 (**4**)	128 (1)
Vancomycin	0	> 256	> 256	> 256
	MIC/8	> 256 (—)	> 256 (—)	> 256 (—)

MIC: Minimal Inhibitory Concentration; Cl_2CH_2/MeOH: Dichloromethane/methanol; MeOH: Methanol; AME: Antibiotic-modulating effects; —: MF not determined; Values in bold represent modulation factor ≥ 2.
